# Psychometric limitations of the 13-item Sense of Coherence Scale assessed by Rasch analysis

**DOI:** 10.1186/s40359-017-0187-y

**Published:** 2017-06-08

**Authors:** Anners Lerdal, Randi Opheim, Caryl L. Gay, Bjørn Moum, May Solveig Fagermoen, Anders Kottorp

**Affiliations:** 1 Department of Nursing Science, Institute of Health and Society, Faculty of Medicine, University of Oslo, Blindern, Postbox 1130, 0318 Oslo, Norway; 2 Department for Patient Safety and Research, Lovisenberg Diakonale Hospital, Nydalen, Postboks 4970, 0440 Oslo, Norway; 3 Department of Gastroenterology, Division of Medicine, Oslo University Hospital, Nydalen, P.O. Box 4956, 0424 Oslo, Norway; 4 Department of Family Health Care Nursing, School of Nursing, University of California, San Francisco, 525 Parnassus Ave, San Francisco, 94143 CA USA; 5 Institute of Clinical Medicine, University of Oslo, Blindern, P.O. Box 1171, 0318 Oslo, Norway; 6 Department of Occupational Therapy, University of Illinois at Chicago, IL, 1200 West Harrison, St. Chicago, 60607 IL USA

**Keywords:** Sense of coherence, Rasch analysis, Psychometrics, Inflammatory Bowel Disease, Validity, Reliability

## Abstract

**Background:**

A person’s sense of coherence (SOC) reflects their perception that the world is meaningful and predictable, and impacts their ability to deal with stressors in a health-promoting manner. A valid, reliable, and sensitive measure of SOC is needed to advance health promotion research based on this concept. The 13-item Sense of Coherence Scale (SOC-13) is widely used, but we reported in a previous evaluation its psychometric limitations when used with adults with morbid obesity. To determine whether the identified limitations were specific to that population or also generalize to other populations, we have replicated our prior study design and analysis in a new sample of adults with inflammatory bowel disease (IBD).

**Methods:**

A sample of 428 adults with IBD completed the SOC-13 at a routine clinic visit in Norway between October 1, 2009 and May 31, 2011. Using a Rasch analysis approach, the SOC-13 and its three subscales were evaluated in terms of rating scale functioning, internal scale validity, person-response validity, person-separation reliability and differential item functioning.

**Results:**

Collapsing categories at the low end of the 7-category rating scale improved its overall functioning. Two items demonstrated poor fit to the Rasch model, and once they were deleted from the scale, the remaining 11-item scale (SOC-11) demonstrated acceptable item fit. However, neither the SOC-13 nor the SOC-11 met the criteria for unidimensionality or person-response validity. While both the SOC-13 and SOC-11 were able to distinguish three groups of SOC, none of the subscales could distinguish any such groups. Minimal differential item functioning related to demographic characteristics was also observed.

**Conclusions:**

An 11-item version of the sense of coherence scale has better psychometric properties than the original 13-item scale among adults with IBD. These findings are similar to those of our previous evaluation among adults with morbid obesity and suggest that the identified limitations may exist across populations. Further refinement of the SOC scale is therefore warranted.

## Background

Sense of coherence (SOC) is the core concept in the salutogenic theory introduced by the medical sociologist Aaron Antonovsky [1]. SOC reflects a person’s resources and dispositional orientation, which enables one to manage tension, reflect on internal and external resources and deal with stressors in a health-promoting manner [2]. Systematic reviews in general populations and in chronic disease groups conclude that SOC is strongly correlated with a person’s mental health [3] and impacts health-related quality of life (HRQoL). SOC comprises three components: a cognitive component (comprehensibility), a behavioral component (manageability), and a motivational component (meaningfulness). Antonovsky theorized that these three components are dynamically interrelated [1]. Furthermore, he proposed that the “strength of one’s SOC [is] a significant factor in facilitating the movement toward health” [4]. Studies report that SOC is associated with health behavior [5, 6] and is also a suitable outcome variable for patient education courses [7, 8].

SOC has been studied worldwide in a number of different populations including patients with somatic and mental health problems, and in different age groups in the general population [9]. IBD is a chronic, relapsing inflammation of the gastrointestinal tract, with common symptoms including abdominal pain, tenesmus, frequent and urgent diarrhea, as well as general symptoms like fever and weight loss [10]. Patients diagnosed with IBD face the prospect of a lifelong medical condition with a heterogeneous, unpredictable and potentially debilitating disease course [10]. IBD is associated with psychological stress, depression and anxiety as well as increased risk of psychological comorbidities [11, 12]. The disease often imposes a considerable symptom burden and significantly impacts the patient’s daily life and HRQoL [13].

SOC is typically measured using the specifically designed SOC instrument [1]; the widely used 13-item version (SOC-13) is an abbreviation of the original 29-item instrument (SOC-29). Since the anchors of each item are different, a short instrument is warranted, particularly from a feasibility point of view. The psychometric properties of the SOC-13 have primarily been evaluated with classical statistical methods (i.e., Cronbach’s alpha, inter-item correlation and factor analysis) and in general populations of students [14] and active older people, as well as patients with chronic illnesses such as cancer [15] or cardiac disease [16]. The studies have generally concluded that the SOC-13 is a reliable and valid instrument. However, the Rasch measurement model from modern test theory has certain advantages over more classical approaches because Rasch models provide a more in-depth evaluation of individual items and person patterns of responses. The modern test theory approaches also support exploring current validity evidence based on internal structure and response processes [17]. Thus, numerous established instruments are now being re-evaluated using Rasch models (e.g. [18, 19]), and assessed and compared in different populations [20–22]. The in-depth evaluation may also provide important information about the substantive, content, structural, and external validity and generalizability of the instrument [20, 23].

In a previous study [24], we assessed the psychometric properties of the SOC-13 in a sample of 142 adults with morbid obesity. The study showed that a 12-item version (SOC-12) without item #1 demonstrated better psychometric properties than the original SOC-13. The subscales, in particular Comprehensibility and Manageability, had low person-separation indices, indicating that the scales were not able to separate these persons into at least two groups. As these findings were investigated in a sample of people with morbid obesity and generally low SOC scores on a waiting list for bariatric surgery [7], the study findings may not generalize beyond that specific population. Findings reported by Naaldenberg et al. [25] in a community dwelling population of older adults showed that an 11-item version (SOC-11) without items #2 and #4 demonstrated better psychometric properties than the 13-item version and indicated substantial differences in the psychometric properties of the scale with regards to differences in populations.

In light of these differing findings, it is crucial to explore whether similar patterns in the SOC scores exist across different client groups, indicating empirical support for a generic theoretical structure. Thus, the aim of this study was to assess the psychometric properties of the SOC-13 in a sample of adults with inflammatory bowel disease (IBD) to determine whether they differ from or replicate our prior findings in adults with morbid obesity. Using a similar analytic approach as our prior study, we aim to evaluate: 1) the functioning of the rating scales, 2) the fit of the SOC items to the Rasch model, 3) unidimensionality, 4) person-response validity, 5) measurement precision, as demonstrated by the ability of the subscales to separate the sample into distinct strata, and (6) differential item functioning (DIF) in relation to socio-deomographic variables (i.e., age, gender, civil status, education and work status).

## Methods

### Study design and data collection

Patients attending hospital outpatient clinics in Norway (listed under Acknowledgements) were consecutively invited to participate in the study from October 2009 through May 2011. Eligible patients were ≥ 18 years of age and had a previously verified IBD diagnosis of either ulcerative colitis (UC) or Crohn’s disease (CD). After providing informed consent, participants were asked to fill out a questionnaire during the clinic visit. If preferred, participants could complete the questionnaire at home and return it by mail (prepaid). Thirty of the 460 consenting patients did not return the questionnaire and two patients did not complete the SOC questionnaire (*N* = 428, response rate 93%). Further details regarding the data collection have been previously published [26, 27].

### Study site

The study recruited patients with IBD who attended outpatient clinics at hospitals in eastern, western, and southern Norway between October 1, 2009 and May 31, 2011.

### Measurements

Socio-demographic data was self-reported and included age (<40 vs. ≥40 years), gender, civil status (married or cohabitant vs. not), educational level (≤12 vs. >12 years of education), and work status (working, including being a student vs. not working, including being a pensioner or disabled).

Sense of coherence was measured with the Norwegian version of the SOC-13 [1], which consists of 13 items rated on a 7-point Likert scale. In addition to the SOC-13 total scale, it has three subscales: Meaningfulness (4 items), Comprehensibility (5 items), and Manageability (4 items). In addition, self-reported data were collected on the participant’s use of complementary and alternative medicine, HRQoL, fatigue, and generalized self-efficacy. Disease data were collected from their medical records.

### Statistical analysis

As in similar previous studies [28], a Rasch model was chosen to analyze the SOC subscales as the items are intended to represent different aspects of the sense of coherence that are assumed to vary in challenge among adults with IBD. The Rasch model takes each item score and adjusts the final person measure based on relative differences in item challenge [29–31].

A Rasch model analysis converts the pattern of raw ordinal scores from the SOC items into equal-interval measures. This process is performed using a logarithmic transformation of the odds probabilities of responses of the SOC items. The Rasch analysis also provide various statistical outputs used to examine whether items from a scale measure a unidimensional construct [29, 32]. If the data supports.evidence of internal structure and unidimensionality, the converted responses from the SOC can be used as valid measures of sense of coherence. This transformation simultaneously results in a measure of each person’s sense of coherence, as well as a measure of challenge for each of the items along the same calibrated continuum (from a low sense of coherence [items relatively easy to agree with] to a high sense of coherence [items relatively challenging to agree with]). Although the SOC uses a generic rating scale from 1 to 7, the scale is formulated differently across items and therefore may not function in a similar manner across all items. For example, item #2 asks ‘*Has it happened in the past that you were surprised by the behaviour of people whom you thought you knew well?*’, with response alternatives ranging from: 1 = ‘*never happened*’ to 7 = ‘*always happened*’, while item #4 states ‘*Until now your life has had…*’ with response alternatives ranging from: 1 = ‘*no clear goals or purpose at all*’ to 7 = ‘*very clear goals or purpose*’. Therefore a partial credit model, developed for scales where ratings may differ across items, was applied to the SOC in this analysis. The WINSTEPS analysis software program, version 3.69.1.16 [31] was used to conduct the Rasch analyses in this study.

This study was designed with 6 steps to evaluate validity evidence based on response processes, internal structure, and precision of the generated measures [17]. In step 1, the functioning of the rating scales used in the SOC (evidence based on response processes) was evaluated according to the following criteria: a) the average measures for each step category on each item should advance monotonically, and b) a criterion less than 2.0 was expected in outfit mean square *(MnSq)* values for step category calibrations [33, 34]. In step 2, the fit of the items to the Rasch model was then analyzed (evidence based on internal structure). Step 3 consisted of a principal component analysis to evaluate unidimensionality (evidence based on internal structure), step 4 addressed aspects of person-response validity SOC (evidence based on response processes), step 5 assessed person-separation reliability (precision of the generated measures), and step 6 evaluated differential item functioning (DIF) in relation to socio-demographic variables.

*Evidence based on internal structure* (step 2) and *evidence based on response processes* (step 4) were investigated using item and person goodness-of-fit statistics using the WINSTEPS program to generate mean square *(MnSq)* residuals and standardized *z-*values. These measures indicate the degree of match between actual responses on the SOC items and the expected responses based upon the assertions stated in the Rasch model. We chose *infit* statistics to evaluate goodness-of-fit across individual items and across persons in this study [29, 35], using a sample-size adjusted criterion for *item goodness-of-fit* set for infit *MnSq* values between 0.7 and 1.3 logits [36].

The criterion for evaluating *evidence based on person response processes* was to accept infit *MnSq* values ≤ 1.4 logit and/or an associated *z* value < 2 [37, 38]. It is generally accepted that 5% of the sample, by chance, may not demonstrate acceptable goodness-of-fit without a serious threat to person-response validity [37, 38].

To explore the presence of additional explanatory dimensions in the data (evidence based on internal structure), a *principal component analysis* (PCA) of residuals was performed to evaluate the unidimensionality of each of the SOC subscales (step 3) [31]. The criterion for unidimensionality was that at least 50% of the total variance should be explained by the first latent dimension [39, 40].

To further determine whether the SOC could differentiate people with different levels of SOC, the *person-separation reliability index* was calculated (step 5). For a scale to distinguish between at least two distinct groups, an *index* of 1.5 is required.

Given that Antonovsky developed the SOC scale based on his salutogenic theory, we initiated the process described above by examining each of the SOC subscales (Meaningfulness, Comprehensiveness, and Manageability). If the data did not meet the various criteria that were set, we used the following approach. First, if the rating scale did not function according to the set criteria, we collapsed the disordered scale steps so that the rating scale met the criteria [31]. Then, if an item did not demonstrate acceptable goodness-of-fit to the model, it was removed and the psychometric properties were re-analyzed with the remaining items. This procedure was repeated until all items demonstrated acceptable goodness-of-fit. Next, unidimensionality, person goodness-of-fit, and person reliability index were examined. Because the SOC scale is used to generate a total score in addition to the subscale scores, we also examined the SOC total scale using similar steps and procedures as described for the 3 subscales.

SPSS for Windows Version 22.0 software (IBM Corp., Armonk, NY, USA) was used to describe the sample’s demographic characteristics.

## Results

### Sample characteristics

Of the 428 patients, 190 (44%) had UC and 238 (56%) had CD. The sample had a mean age of 40.8 ± 12.3 years (range 18 to 79 years) with 210 (50.4%) under 40 years of age, 212 (49.5%) were women, 309 (72%) were married, 282 (66%) were in paid work or in school, and 200 (47%) had more than 12 years of formal education. Median disease duration was 9 years (range 0.1 to 45 years) and the majority of patients (*n* = 257, 60%) reported having active disease at the time of the study.

### Rating scale functioning (step 1)

When evaluating rating-scale function of the SOC subscales, items #5, #7and #12 did not meet the set criteria (See Table 1). The average step calibration measures did not advance monotonically in the following items: scale step categories 1 and 2 were reversed in items #7 and #12 in the Meaningfulness subscale, and scale steps 1, 2, and 3 were reversed in #5 in the Manageability subscale. The remaining ten items demonstrated acceptable values. We therefore collapsed the scale step categories that were reversed in these items before proceeding to the other analyses.

**Table 1:** Rasch analysis of the psychometric properites of the Sense of Coherence (SOC) subscales, total scale, and reduced scale (*N* = 428)

Step		Meaningfulness subscale (4 items) (#1, #4, #7, and #12)	Comprehensibility subscale (5 items) (#2, #6, #8, #9, #11)	Manageability subscale (4 items) (#3, #5, #10 and #13)	Total scale (13 items)	Reduced scale (11 items) (#1 and #5 omitted)
1	Items not meeting criteria for rating scale	#7 and #12 ^a^	None	#5^b^	#7 and #12 ^a^; #5 ^b^	#7 and #12 ^a^
2	Item misfit	None	None	None	#1 and #5 ^c^	None
3	Variance explained by1^st^ dimension, %	55.0%	50.1%	47.3%	39.7%	42.8%
	2^nd^ dimension, %	16.1%	14.6%	21.2%	9.6%	8.5%
4	Person misfit, *n* (%)	15 (3.5%)	19 (4.4%)	20 (4.7%)	40 (9.6%)	29 (6.8%)
	Maximum score, *n* (%)	19 (4.4%)	7 (1.6%)	12 (2.8%)	2 (0.5%)	2 (0.5%)
	Minimum score, *n* (%)	None	None	None	None	None
5	Person-separation index (without extremes)	1.41	1.54	1.18	2.19	2.10
	Cronbach alpha	0.67	0.70	0.58	0.83	0.82
6	Differential item functioning (DIF)					
	Age (<40 years vs ≥40 years)	No DIF	No DIF	No DIF		
	Gender (male vs female)	No DIF	No DIF	No DIF		
	Civil status (married/cohabitant vs not)	No DIF	#6 ^d^	No DIF		
	Education (≤12 years vs >12 years)	No DIF	No DIF	No DIF		
	Work (Working/student vs not)	No DIF	No DIF	No DIF		

^a^#7 and #12: Scale step categories 1 and 2 reversed. After collapsing scale step categories 1 and 2, the rating scale met the criteria set

^b^#5: Scale step categories 1 to 3 disordered (3,2,1,4,5,6,7). After collapsing scale step categories 1 to 3, the rating scale met criteria set

^c^#1: Infit MnSq 1.32 StdZ 3.7; #5: Infit MnSq 1.34 StdZ 4.1

^d^Group 1: 46.61 Group 2: 44.11 p < .01

### Item goodness-of-fit and unidimensionality for the SOC subscales (steps 2 and 3)

**Fig. 1:**
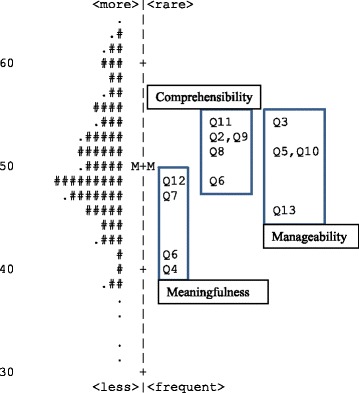
Item hierarchy for subscales of the SOC. Scoring of items: 2, 3, 7, and 10 are reversed

In the analysis of the SOC subscales, all items demonstrated acceptable goodness-of-fit to the Rasch model. The continuum of challenge calibrations of the SOC items is presented in Fig. 1. The PCA for the SOC subscales is presented in Table 1. The Rasch model explained between 47.3 to 55.0% of the total variance in the dataset across the subscales. Therefore, evidence of internal scale validity was acceptable for the Meaningfulness and Comprehensibility subscales, but mixed for the Manageability subscale.

### Person goodness-of-fit and reliability for the SOC subscales (steps 4 and 5)

Of the 428 SOC surveys, 3.5 to 4.7% of the participants did not demonstrate acceptable goodness-of-fit to the Rasch model, depending on the subscale. The number of participants with maximum and minimum scores (ceiling and floor effects) across the SOC subscales are shown in Table 1. As none of the subscales demonstrated more than 4.4% maximum or minimum scores, this was not considered a threat to target validity.

The person separation index for the SOC subscales ranged from 1.18 (Manageability) to 1.54 (Comprehensibility), with the latter being the only subscale sensitive enough to detect the minimum of two distinct strata in the sample.

### Differential item functioning (step 6)

Analyses of DIF of the SOC items in relation to the socio-demographic variables revealed no DIF for any of the items in relation to age, gender, education or work status. The only identified DIF was in relation to civil status on item #6 (*Do you have the feeling that you are in an unfamiliar situation and don’t know what to do?*); the item was relatively easier to agree with for people who were not married/cohabitant compared to the other items.

As the results of the SOC subscales generated mixed evidence of validity and reliability, we continued our analysis to examine the SOC total scale. In particular, the separation indices for the Meaningfulness and Manageability subscales were lower than 1.5, which indicates that these scales were not able to distinguish any distinct strata in the sample and were therefore not functioning as reliable scales.

### SOC total scale (steps 2 through 5)

In the analysis of the SOC total scale, all but two items (#1 and #5) demonstrated acceptable goodness-of-fit to the Rasch model. The Rasch model explained 39.7% of the total variance in the dataset. Therefore, evidence of unidimensionality was also mixed for the SOC total scale. The proportion of participants that did not demonstrate acceptable goodness-of-fit to the Rasch model was 9.6% in the SOC total scale with a separation index of 2.19, which indicates that three levels of SOC could be distinguished in the sample.

As items #1 and #5 did not meet the criteria for item fit, we excluded these items and re-analyzed the SOC total scale with the remaining 11 items (SOC-11). All of the SOC-11 items demonstrated acceptable goodness-of-fit to the Rasch model, the explained variance was actually slightly higher than in the SOC-13, the proportion of person misfit was slightly reduced, and the person separation index for the SOC-11 was only marginally reduced compared to the SOC-13 (See Table 1).

In Fig. 1, the items of the SOC-13 are presented along a linear continuum. The items in the Meaningfulness subscale are at the lower end of the continuum, indicating that these items are generally easier to agree with and therefore may be more fundamental to the concept of SOC as compared to the other subscales.

## Discussion

Our evaluation of the SOC-13 in a population of adults with IBD is a replication of our prior psychometric evaluation of the SOC-13 in a sample of adults with morbid obesity, which yielded similar findings. In the present study, the SOC-13 did not meet our criteria for item scale validity, as two items did not fit with the Rasch model (items #1 and #5). However, an 11-item version (SOC-11) omitting those two items showed satisfactory internal scale validity in adults with IBD. In terms of person-response validity, each of the three subscales met the set criteria, but although the SOC-11 was slightly better than the SOC-13, neither of the total scales met the set criteria. The person-separation reliability was satisfactory at the group level for both the SOC-13 and SOC-11, as both scales could distinguish three groups. However, two of the three subscales (Meaningfulness and Manageability) could not separate the responses into groups, which limits their usefulness.

The psychometric limitations of the SOC-13 identified in this study of adults with IBD are similar to those identified in our prior study among adults with morbid obesity. These replicated findings raise some concerns related to the SOC-13 that may apply regardless of the population being studied. We found one other recent study which has tested the psychometric properties of the SOC-13 by Rasch analysis in a sample of healthy adults [41]. Similar to our findings, the scale steps of some of the items did not advanced monotonically and had to be collapsed. Furthermore, one item (item #1) showed misfit, and consistent with the separation index determined in our study, the scale could separate the sample into three different levels of SOC. Thus, the findings generated from a series of studies in various samples/populations share some generic limitations found in the SOC scale.

First, relying on a 7-category rating scale to produce more precise estimations of sense of coherence is not supported by the empirical findings. Instead, a five or six category scale seems to support more distinct categories of the target concept. Similar findings have also been found among both healthy people as well as chronic patients [24, 41].

Second, a lack of unidimensionality seems to be present in the Manageability subscale as well as the SOC total scale. Even though it can be conceptually acceptable that the theoretical concepts in a psychological model are not clearly distinct from each other, it creates a challenge when aiming to measure such constructs in a precise and valid manner. A prior study in a community-dwelling older population found that an 11-item version of the SOC had better psychometric properties than the SOC-13 [25]. These findings, combined with those from our current study and our previous study among adults with obesity [24], constitute growing evidence that the SOC-13 lacks internal scale validity, unless specific actions are taken, such as deleting item #1 from the scale. Item #1 seems to misfit the Rasch model across various groups, and therefore supports a more generic conclusion that this item does not fit the underlying sense of coherence construct. Future studies should explore the internal scale validity of the original SOC-29 as the generic findings of the SOC-13 do not support a unidimensional construct, unless items are deleted.

Third, the relative lack of precision, assessed in this study by the person separation index, needs to be considered, especially for the SOC subscales. However, when the subscales are combined into one total score, they are better targeted to the sample (See Fig. 1) and also generate more precise measures of the construct (See Table 1). Both the SOC-13 and the reduced SOC-11 versions were able to detect three distinct groups of SOC. Although this can be relevant for group comparisons, some concerns should be raised in using the SOC as an outcome measure on an individual basis. Moreover, the measures are likely not precise enough to detect small but potentially important changes over time or in relation to intervention. It may also be notable that the classical Cronbach alpha values reported for the SOC scales are not sensitive to detect item misfit or lack of separation, which supports the use of several methodological approaches derived from both classical and modern test theory in evaluating evidence for the validity of clinical scales.

### Study limitations

The current study has some limitations. An even larger sample would have allowed more in-depth analysis of subgroups, e.g., whether people demonstrating unacceptable goodness-of-fit share some unique characteristics. In addition, this analysis was based on a sample of Norwegian persons with IBD, and therefore it may not be evident whether the findings are specific to those with IBD, the Norwegian version of the SOC, or a combination of both. An earlier published Norwegian study with people with morbid obesity demonstrate some similar findings as in this study, indicating that the findings may be generic and not limited to a specific diagnosis. Finally, future studies that include both classical and modern test theory would be helpful for discerning whether differing psychometric findings are due to the different approaches or simply reflect differing samples.

## Conclusions

Findings from this and other studies performed in a Scandinavian context indicate that the SOC-13 does not meet criteria for validity or precision in various samples. This raises concern about using the sum scores of the SOC scale and subscales as valid measures of the target phenomenon, as the raw sum scores do not fully represent variations in a sample. The degree of challenge for each item should be taken into consideration in estimations of individual measures of sense of coherence, or transformation tables should be developed. Future research should focus on developing a better version of the SOC scale based on item response theory models, starting with the SOC-29 item pool to develop and evaluate both subscales and total scales. Reduction of the rating scale should also be considered, as the current 7-point scale does not function as an interval scale.

## Abbreviations

DIF: Differential item functioning
IBD: Inflammatory bowel disease
PCA: Principal component analysis
SOC: Sense of coherence
